# Therapeutic and legal aspects of psilocybin in cancer-related depression

**DOI:** 10.3389/fpsyt.2025.1591864

**Published:** 2025-08-04

**Authors:** Małgorzata Wierzbicka, Renata Kopczyk, Aleksandra Gerlach, Joanna Rymaszewska

**Affiliations:** ^1^ Research and Development Center, Regional Specialist Hospital, Wrocław, Poland; ^2^ Institute of Human Genetics, Polish Academy of Sciences, Poznań, Poland; ^3^ Faculty of Medicine, Wroclaw University of Science and Technology, Wrocław, Poland; ^4^ Head of Department of Clinical Neurosciences, Faculty of Medicine, Wroclaw University of Science and Technology, Wrocław, Poland; ^5^ Faculty of Management, Wroclaw University of Science and Technology, Wrocław, Poland

**Keywords:** head neck cancer, treatment, distress, depression, antidepressant utilization, psilocybin, legislation

## Abstract

Depression prevalence is markedly elevated in oncological patients, particularly among head and neck cancer (HNC) cohorts, who face twice the prevalence of major depressive disorder (MDD) compared to other cancer populations. MDD in this context independently predicts poorer clinical outcomes and increased morbidity. HNC management often involves acute surgical interventions with disfiguring effects, creating a narrow therapeutic window for conventional antidepressants requiring weeks to achieve efficacy. Psychological interventions face similar time constraints, complicating perioperative mental health support. Psilocybin – metabolized to psilocin – modulates serotonin (5-HT2A) and dopamine receptors, demonstrating rapid antidepressant effects within hours rather than weeks. Clinical trials validate its superiority over escitalopram in MDD treatment and efficacy in PTSD and treatment-resistant depression. Despite these benefits, no studies explore perioperative applications in HNC patients. Psilocybin lacks international scheduling under UN conventions, permitting variable national policies: Australia - MDMA/psilocybin prescriptions (2023), USA - Insurance billing codes (2024), Portugal - Decriminalized, South Africa - Prescription medicine. In Polish Context psilocybin remains restricted to research settings, classified as a Group I-P substance under the 1971 Psychotropic Convention. This legal framework complicates clinical implementation despite emerging evidence of therapeutic potential. The critical challenge lies in reconciling psilocybin’s rapid antidepressant properties with regulatory barriers, particularly for HNC patients requiring immediate psychiatric support post-surgery. Interdisciplinary collaboration between oncologists, psychiatrists, and policymakers is essential to design ethical clinical pathways under current legislative constraints.

## Introduction

Depression demonstrates markedly elevated prevalence among oncological patients compared to the general population, with particularly striking manifestations in head and neck cancer (HNC) cohorts (1–4). Empirical evidence indicates that HNC patients exhibit twice the likelihood of screening positive for depression on validated assessment tools relative to other cancer populations, despite comparable rates of self-reported depressive symptoms and antidepressant utilization (5). The distinctive characteristics of HNC management, encompassing urgent and potentially disfiguring surgical interventions, inherently precipitate reactive depression during the postoperative phase, while simultaneously presenting challenges for achieving therapeutic antidepressant levels.

Contemporary medicine is relentlessly pursuing more effective therapeutic approaches in the battle against depression, a condition fundamentally associated with dysregulation of neurotransmitters, particularly serotonin and noradrenaline as well as immune and hormone systems (6). Psilocybin, a naturally occurring psychoactive compound, has emerged as a promising subject of extensive clinical investigation for its profound influence on perceptual processing, cognitive function, and emotional state.

Thus, the crucial question remains how to legally implement psilocybin therapy as an interventional treatment in a hospital setting for patients following ablative head and neck cancer surgery.

## Methods

Systematic approach to literature search encompass a literature review of subject matter characterized by complex methodology due to the interdisciplinary nature of publications. Database Selection was a first step to utilize multiple academic databases including PubMed, Scopus, and Embase for medical sciences (7, 8). The search process begins with identifying core concepts from research questions, developing synonyms and alternative terms, using controlled vocabulary and thesaurus terms (9, 10).

Two primary approaches enhance search depth by forward citation searching, i.e finding articles that have cited key references and backward citation searching, i.e. examining reference lists of relevant articles (11). Block search method involves dividing research questions into distinct conceptual blocks, organizing search terms within each block, combining blocks using Boolean operators (12). First bloc covers the cancer treatment area, second bloc concern depression and potential psychedelic intervention. Third bloc of documents consist of legal acts work-up. Refinement process and quality control ensure search comprehensiveness by validating search strategies with peer review and testing searches against known relevant articles (13).This systematic approach ensures comprehensive coverage of the literature while maintaining scientific rigor and reproducibility (10).

## Results

### Special considerations for tailored antidepressant application in HNC

A unique application concerns patients undergoing extensive HNC surgeries. On one hand, it is the treatment of choice, yet on the other hand, these amputation procedures, despite advanced reconstruction techniques, are mutilating and permanently alter a person’s visage. Additionally, the compressed temporal window between surgical qualification and intervention, typically spanning less than twenty-one days, fundamentally precludes the establishment of efficacious antidepressant pharmacodynamics. Furthermore, the phenomenon of depression self-reporting in HNC patients’ population frequently results in substantial underestimation and subsequent inadequate therapeutic intervention (5). Moreover, these patients frequently manifest alcohol withdrawal syndromes, which amplify anxiety states and potentially precipitate aggressive behavioral manifestations (5). Severe postoperative depression significantly impairs essential aspects of recovery, including healthcare provider collaboration, therapeutic protocol adherence, early rehabilitation implementation, and optimal wound healing conditions. Thus, preventing depression during cancer treatment may be of great benefit (2), both by psychological intervention methods (14) and pharmacotherapy (4, 15).

Well-being of oncological patients have recently emerged as a prominent subject of consideration in the literature. Patients who undergo total laryngectomy (TL) experience significant psychological disturbances that manifest with varying intensity and timing following surgery (16–19). Overall, severe depressive disorders occur in 12-63% of patients, with depressive mood presents in even higher percentages (17, 20), 63% of patients without disorders experience a physiological depressive reaction, while 26% exhibit psychological disturbance (20). Notably, 6% of patients developed psychological disorders in the first year following laryngectomy (21). Women and men undergoing TL generally suffer from psychological disorders at similar rates (23% vs 37% at 3 months and 22% vs 21% at 12 months post-surgery). However, women more frequently experience post-traumatic stress disorder and late generalized anxiety disorders (21). Patients under 65 years of age show a greater propensity for psychological problems. Sociodemographic factors, such as low income, lack of stable employment, and unemployment, also increase the risk of depression (20, 22).

### Therapeutic potential of psilocybin

The pharmacological mechanism of action of psylocybin is attributed to structural homology with serotonin, enabling selective binding to specific serotonergic receptors within the central nervous system. Following administration, psilocybin (4-phosphoryloxy-N,N-dimethyltryptamine), an indoleamine pro-drug, undergoes enzymatic dephosphorylation to yield psilocin, the primary pharmacologically active metabolite. Psilocin demonstrates uniform biodistribution throughout the organism, with particular tropism for key cerebral regions: the neocortex, mediating cognitive processes including learning, memory consolidation, and interpretative functions, the thalamus, orchestrating sensory signal processing, the hippocampus, a critical component of the limbic system governing emotional regulation. Upon reaching its central targets, it modulates serotonergic neurotransmission, modulate medial prefrontal cortex (mPFC) and its cortico-limbic functional networks and precipitating alterations in consciousness and perceptual processing. The idea that psilocybin is a psychoplastogen—one that promotes brain plasticity to change neural connections and, eventually, behavior—is supported by encouraging results from recent research investigating the synaptogenic effects of psilocybin (23).

Psilocin influences glutamate and GABA release in the nucleus accumbent, hippocampus and amygdala, increases ACh levels in the hippocampus leading to anxiolytic effects and stimulating sensorimotor and perceptual functions (24–26). Pharmacologically, it resembles LSD but exhibits approximately 100 times weaker effects. After ingestion, psilocybin appears in serum within 20–40 minutes, with initial effects manifesting after 15–40 minutes, and the effects typically lasting 4–6 hours maximum. The individualized response to psilocybin depends on multiple factors including metabolism efficiency, body weight, prior food consumption, concurrent use of alcohol or other psychoactive substances, and the patient’s mood at the time of administration.

### Therapeutic applications of psylocybin

Psilocybin has demonstrated remarkable therapeutic potential in treating a broad spectrum of psychiatric and behavioral disorders. Clinical studies have provided evidence for the compound’s efficacy in the treatment of post-traumatic stress disorder (PTSD), treatment-resistant depression, and major depressive disorder (MDD) (6). Furthermore, psilocybin has proven beneficial in alleviating anxiety and psychological distress in oncological patients, as well as reducing end-of-life anxiety in terminally ill individuals (23). In cases of nicotine and alcohol dependence, impressive abstinence rates of up to 80% have been documented for periods extending to six months in smoking cessation. These promising outcomes open new perspectives in the fields of psychiatry and addiction medicine.

Psylocibin does not induce physical dependence, though it may lead to psychological dependence, and due to its potent euphoric effects, this substance carries a high potential for abuse. For these reasons, it is classified in Schedule I-P, designating compounds with no medical application and high abuse potential. Psilocybin is excluded from pharmaceutical distribution and may only be utilized in scientific research. Nevertheless, combining the themes discussed in the introduction and summarizing the issues at hand, psilocybin represents an ideally suited compound for HNC patients and can be considered as a useful treatment method.

### The global evolution of psychedelic drug policy

The worldwide criminalization of psychedelics began in 1966 when the United States initiated restrictions on LSD possession, sale, and production. The Staggers-Dodd Act of 1968 federally prohibited psilocybin mushrooms, followed by the 1970 classification of psilocybin as a Schedule I controlled substance, indicating no accepted medical use and high abuse potential. The UN Convention on Psychotropic Substances of 1971 further prohibited these substances globally. Currently, plants containing psilocybin and psilocin are not controlled under international conventions, leaving enforcement to national legislation. Portugal pioneered decriminalization in 2001, treating all drug possession as administrative rather than criminal violations. Denver, Colorado made history in 2019 by becoming the first U.S. city to decriminalize psilocybin mushrooms, with Oregon following in 2020 by both decriminalizing and regulating their use.

Several countries have embraced medical applications: South Africa and Australia recognize psychedelics as prescription medicines, Jamaica, Samoa, and Nepal maintain legal status for psilocybin, Portugal, Switzerland, Spain, and Canada have decriminalized usage. The U.S. will implement medical billing codes for psychedelic therapy in 2024, just after Australia permits MDMA and psilocybin prescriptions since July 2023. The UK leads in innovation, funding psychedelic research with £1.5 million for ketamine therapy studies and granting innovation passports for MDMA-assisted PTSD therapy, COMPASS Pathways’ psilocybin therapy, Small Pharma’s DMT treatment for major depressive disorder.

The legal regulations concerning psilocybin in Poland are outlined in the Act of July 29, 2005, on Counteracting Drug Addiction (27) – hereinafter referred to as the “CDA.” Additionally, international law regulations apply to this matter. In this context, it is necessary to refer to the provisions of the Convention on Psychotropic Substances of February 21, 1971 (28) – hereinafter referred to as the “Convention,” ratified by the People’s Republic of Poland in August 1976 (29). The provisions of the CDA are consistent with the international regulations set forth in the Convention. Psilocybin is listed in Schedule I of the Convention (see the List of Substances Included in Schedule I). Importantly, pursuant to Article 7 of the Convention.

Under Article 32(1) of the CDA, the legislator, based on the provisions of the Convention, divided psychotropic substances into four groups designated as: I-P, II-P, III-P, and IV-P. The classification criteria are based on two main factors: the degree of addiction risk when used for purposes other than medical and the extent of medical application of psychotropic substances. Notably, psychotropic substances in groups II-P, III-P, and IV-P can only be used for medical, industrial, or research purposes, while those in group I-P can only be used for research purposes (Article 33 of the CDA). The list of psychotropic substances classified by group is provided in Annex 1 to the Regulation of the Minister of Health of August 17, 2018, on the list of psychotropic substances, narcotic drugs, and new psychoactive substances (30). Psilocybin is classified under group I-P, which means it can, in principle, only be used for research purposes.

## Discussion

Human clinical research shows that psilocybin causes both short-term, very rapid as well as long-term changes in behavior, mood, and cognition, accompanied by changes in regional activity and connectivity in networks linked to depression. These attributes can be particularly useful in overcoming emotional crises after surgery in severely ill head and neck cancer patients.

Major depressive disorder develops in up to half the patients receiving primary therapy for HNC, resulting in significant morbidity; therefore, preventing depression during cancer treatment may be of great benefit (3). Psilocybin represents an ideally suited compound for HNC patients, who often demonstrate severe tension, distress, anxiety, depression, and are at risk for suicide. Moreover, a significant proportion of patients concurrently experience withdrawal syndrome, as alcohol and tobacco are the primary etiopathogenic factors in these cancers. The crucial question remains how to legally implement psilocybin therapy as an interventional treatment in a hospital setting for patients following ablative head and neck cancer surgery.

There are no reports in the literature regarding the use of psilocybin in the perioperative period, although isolated studies exist concerning depression treatment. In a phase 2, double-blind, randomized, controlled trial involving patients with chronic, moderate-to-severe major depressive disorder (MDD), secondary outcomes generally demonstrated superiority of psilocybin over escitalopram (31).

Unfortunately, legal acts prohibit psychedelic substances usage in medical research in Poland. There are some main provisions. The list of controlled substances is regulated by the Minister of Health’s Regulation of 17 August 2018 regarding psychotropic substances, narcotic drugs, and new psychoactive substances. These substances are categorized based on: addiction risk potential for non-medical use, scope of medical applications. Most psychedelic substances are classified in Group I-P, which has no medical applications, shows high abuse potential, is excluded from pharmaceutical trade, may only be used for scientific research. Some psychedelics (salvinorin A, ketamine, 2C-B) belong to Group II-P, allowing for medical, industrial, or research purposes under specific conditions. At this point, it is worth mentioning the specific ethical considerations associated with this group of psychedelic drugs, which is the need to optimize informed consent procedures, with precise information on the possible alteration of the level of consciousness and its consequences. Using the right settings for psychedelic treatments under standard medical supervision has also been considered as a means to enhance safety for patients under altered states of mind (32).

To summarize, the proposed research project could also serve as a basis for submitting *de lege ferenda* proposals to reclassify psilocybin from group I-P to group IV-P, allowing its use exclusively for medical, industrial, or research purposes. Importantly, the research project does not aim to decriminalize psilocybin. As knowledge of the therapeutic benefits and unique effects of psylocibin develops, the goal is to change its classification in light of its anticipated positive properties for intervention therapy in hospital settings for patients who have undergone ablative surgery for head and neck cancer. However, progress in research is needed to establish effects. Ultimately, this might result in the creation of more precise and efficient clinical practices.

## Data Availability

The original contributions presented in the study are included in the article/supplementary material. Further inquiries can be directed to the corresponding authors.
