# Correction: Therapeutic and legal aspects of psilocybin in cancer-related depression

**DOI:** 10.3389/fpsyt.2025.1698185

**Published:** 2025-09-11

**Authors:** Małgorzata Wierzbicka, Renata Kopczyk, Aleksandra Gerlach, Joanna Rymaszewska

**Affiliations:** ^1^ Research and Development Center, Regional Specialist Hospital, Wrocław, Poland; ^2^ Institute of Human Genetics, Polish Academy of Sciences, Poznań, Poland; ^3^ Faculty of Medicine, Wroclaw University of Science and Technology, Wrocław, Poland; ^4^ Head of Department of Clinical Neurosciences, Faculty of Medicine, Wroclaw University of Science and Technology, Wrocław, Poland; ^5^ Faculty of Management, Wroclaw University of Science and Technology, Wrocław, Poland

**Keywords:** head neck cancer, treatment, distress, depression, antidepressant utilization, psilocybin, legislation

In the original article, the author “Małgorzata Wierzbicka” was erroneously listed as “Wierzbicka Małgorzata.” Her name has been updated in the author list and correspondence section.

The article citation now reads:

“Wierzbicka M, Kopczyk R, Gerlach A and Rymaszewska J (2025) Therapeutic and legal aspects of psilocybin in cancer-related depression. *Front. Psychiatry* 16:1591864. doi: 10.3389/fpsyt.2025.1591864”

The copyright statement reads:

“© 2025 Wierzbicka, Kopczyk, Gerlach and Rymaszewska. This is an open-access article distributed under the terms of the Creative Commons Attribution License (CC BY). The use, distribution or reproduction in other forums is permitted, provided the original author(s) and the copyright owner(s) are credited and that the original publication in this journal is cited, in accordance with accepted academic practice. No use, distribution or reproduction is permitted which does not comply with these terms.”

In the **Author Contributions** statement, the initials “WM” have been changed to “MW”.

The original version of this article has been updated.

